# Different growth and metastatic phenotypes associated with a cell-intrinsic change of Met in metastatic melanoma

**DOI:** 10.18632/oncotarget.12221

**Published:** 2016-09-23

**Authors:** Eri Adachi, Katsuya Sakai, Takumi Nishiuchi, Ryu Imamura, Hiroki Sato, Kunio Matsumoto

**Affiliations:** ^1^ Division of Tumor Dynamics and Regulation, Cancer Research Institute, Kanazawa University, Kakuma, Kanazawa 920-1192, Japan; ^2^ Division of Functional Genomics, Advanced Science Research Center, Kanazawa University, Kakuma, Kanazawa 920-0934, Japan

**Keywords:** drug resistance, HGF, malignant melanoma, met, metastasis

## Abstract

A dynamic phenotypic change contributes to the metastatic progression and drug resistance in malignant melanoma. Nevertheless, mechanisms for a phenotypic change have remained to be addressed. Here, we show that Met receptor expression changes in a cell-autonomous manner and can distinguish phenotypical differences in growth, as well as in metastatic and drug-resistant characteristics. In metastatic melanoma, the cells are composed of Met-low and Met-high populations. Met-low populations have stem-like gene expression profiles, are resistant to chemotherapeutic agents, and have shown abundant angiogenesis and rapid tumor growth in subcutaneous inoculation. Met-high populations have a differentiated phenotype, are relatively resistant to B-RAF inhibitor, and are highly metastatic to the lungs. Met plays a definitive role in lung metastasis because the lung metastasis of Met-high cells requires Met, and treatment of mice with the Met-containing exosomes from Met-high cells facilitates lung metastasis by Met-low cells. Clonal cell fate analysis showed the hierarchical phenotypical changes from Met-low to Met-high populations. Met-low cells either showed self-renewal or changed into Met-high cells, whereas Met-high cells remained Met-high. Clonal transition from Met-low to Met-high cells accompanied changes in the gene expression profile, in tumor growth, and in metastasis that were similar to those in Met-high cells. These findings indicate that malignant melanoma has the ability to undergo phenotypic change by a cell-intrinsic/autonomous mechanism that can be characterized by Met expression.

## INTRODUCTION

The incidence of malignant melanoma is increasing faster than that of other solid tumors [1]. The 5-year survival rate is less than 20% for distant-stage metastatic malignant melanoma [2], indicating that metastasis is the main factor in a poor outcome. In addition, the aggressive characteristics of malignant melanoma results in an innate and acquired resistance to chemotherapeutic and molecular-targeted drugs.

Autocrine and paracrine growth factors secreted within a tumor microenvironment participate in metastasis and drug resistance in malignant melanoma [3, 4]. In addition to extrinsic factors in the tumor microenvironment, tumorigenicity and malignant characteristics are regulated by the heterogeneity of tumor cells. Cancer stem-cell models have provided one explanation for the phenotypic and functional heterogeneity in several types of tumors. In malignant melanoma, however, phenotypic heterogeneity among tumorigenic melanoma cells from patients was reversible and not hierarchically organized [5, 6]. Melanoma cells characterized by the histone demethylase JARID1B are more competent in sustaining tumor growth compared with JARID1B-negative populations, whereas JARID1B expression is reversibly turned on and off [7]. Melanoma cells gain resistance to molecular-targeted agents in response to hypoxia [8]. Thus, adaptive phenotypic plasticity and cell-intrinsic heterogeneity are characteristics of malignant melanoma.

Met/hepatocyte growth factor (HGF) receptor mediates tumor cell proliferation, survival, invasion, and metastasis [9–11]. Previous studies have indicated that Met promotes proliferation, survival, and metastasis in malignant melanoma [12–15]. Comprehensive analysis for drug resistance conferred by the interaction between tumor cells and stromal cells has shown that HGF secreted from stromal cells confers resistance/survival in tumor cells against molecular-targeted agents [3, 4]. Recent study has indicated that Met plays a role in premetastatic niche formation in B16-F10 highly metastatic melanoma [16], suggesting an involvement of Met in the phenotypic plasticity and heterogeneity of melanoma. Understanding of the mechanisms by which the malignant characteristics of melanoma cells are regulated by Met could promote an understanding of stage progression and plasticity, and, hence, the design of better therapeutic interventions.

In the present study, we found that there are different populations in metastatic melanoma cells, and these are characterized by different cell-surface Met expressions. Cells with Met-low and Met-high expressions have different profiles in gene expression, tumorigenicity, growth, and metastasis. Cell-surface Met expression was found to be in dynamic equilibrium and regulated by hierarchical and cell-autonomous changes between Met-low and Met-high populations.

## RESULTS

### Stem cell- and differentiation-related gene expressions in Met-low and Met-high populations

**Figure 1:**
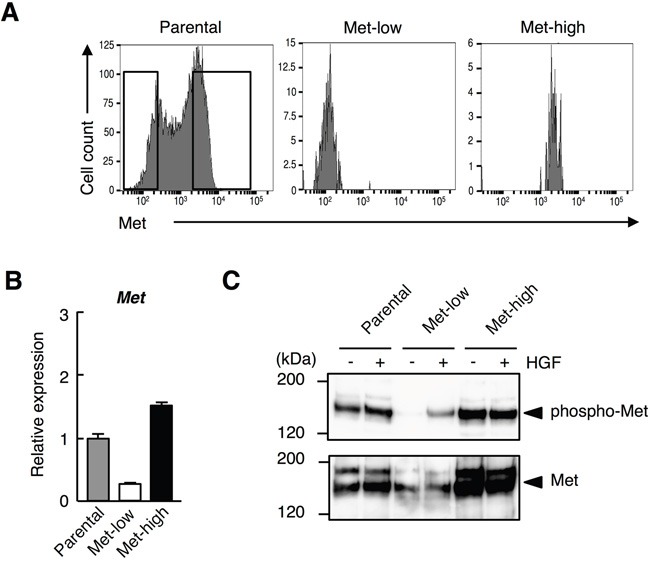
Heterogeneous cell-surface Met receptor expression in B16-F10 melanoma **A.** B16-F10 melanoma cells were stained with anti-Met-PE antibody and analyzed by flow cytometry. Left panel indicates cell-surface Met receptor expression of the unfractionated B16-F10 melanoma cells (parental). Boxes in the panel indicate gates used for cell sorting into Met-low or Met-high. Cell-surface Met expressions of Met-low (middle) and Met-high (right) cells were re-analyzed after sorting. **B.** Expression of *Met* analyzed by quantitative RT-PCR. Following cell sorting, the cells were cultured for 3 days and subjected to quantitative RT-PCR analysis. Each value represents the mean ± SD. The assay was done in triplicate and substantially same results were obtained. **C.** Expression of Met and Met tyrosine phosphorylation. Following cell sorting, the cells were cultured for 2 weeks and subjected to immunoprecipitation and Western blot analysis. In independently performed experiment using another lot Met-low and Met-high cells, substantially the same results was obtained.

The cell-surface Met expression in B16-F10 melanoma cells analyzed by flow cytometer was heterogeneous, and the cells were composed of Met-low and Met-high populations (Figure 1A). We purified Met-low and Met-high cells by cell sorting with > 95% purity (Figure 1A). *Met* mRNA levels were much higher in the Met-high cells than those in the Met-low cells (Figure 1B), suggesting that the difference in cell-surface Met expression was mainly due to a difference in Met gene expression. Met protein levels were higher and Met was phosphorylated in the Met-high cells compared with those in Met-low cells (Figure 1C). Because both Met-low and Met-high cells did not produce detectable levels of HGF, the phosphorylation of Met in Met-high cells seemed to be HGF-independent. HGF stimulated Met phosphorylation in Met-low cells, but this stimulation was not clear in Met-high cells (Figure 1C), while HGF stimulated cell migration of both Met-low and Met-high cells (not shown), suggesting some portions of Met could be activated in a HGF-dependent manner in Met-high cells.

**Figure 2:**
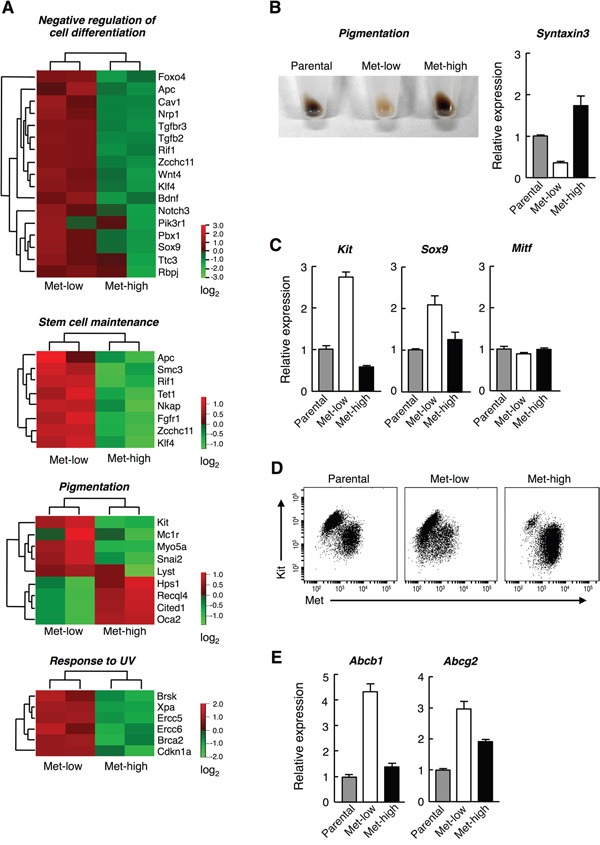
Different characteristics of Met-low and Met-high populations **A.** Expression profiles of genes that functionally belong to “negative regulation of cell differentiation,” “stem cell maintenance,” “pigmentation,” and “response to UV.” The analysis was performed using RNA samples prepared from two independently prepared Met-low and Met-high cells. **B.** Appearance of parental, Met-low and Met-high cells (left), and expressions of *Syntaxin-3* mRNA (right). **C.** Expression of *Kit, Sox9*, and *Mitf* mRNA. **D.** Dual analysis of Kit and Met by flow cytometry. Parental, Met-low, and Met-high cells were stained with anti-Met and anti-Kit antibodies and analyzed by flow cytometry. **E.** Expression of *Abcb1* and *Abcg2* mRNA. Gene expression profiles were analyzed by microarray analysis, and the data obtained by microarray analysis were deposited to the Gene Expression Omnibus and can be accessed by No. GSE69741. Expressions of *Syntaxin-3, Kit, Sox9, Mitf, Abcb1*, and *Abcg2* mRNA were analyzed by RT-PCR. Each RT-PCR analysis were done in triplicate and each value represents the mean ± SD. The same RT-PCR analysis was independently performed twice and substantially the same results were obtained.

To characterize Met-low and Met-high populations, we analyzed gene expression profiles via microarray analysis. Genes differently expressed by more than 2-fold between Met-low and Met-high populations were selected: 886 genes were higher in Met-low than in Met-high cells, while 353 genes were higher in Met-high than in Met-low cells (Supplementary Tables S1, S2). Gene ontology enrichment analysis revealed different expressions of gene clusters between these populations. The gene expressions clustered as “negative regulation of cell differentiation,” “stem cell maintenance,” and “response to UV” were higher in Met-low than in Met-high populations. In contrast, the gene expressions clustered as “pigmentation,” and “melanocyte differentiation” were higher in Met-high than in Met-low populations (Figure 2A, Supplementary Tables S3, S4). In agreement with this, Met-high cells were highly pigmented, whereas Met-low cells were scarcely pigmented (Figure 2B). Likewise, mRNA for *Syntaxin-3*, a key gene for pigmentation of melanosome [17], was expressed at a much higher level in Met-high cells than in Met-low cells (Figure 2B).

Among the gene clusters shown in Figure 2A, *Kit, Snai2*, and *Sox9* are expressed in the progenitor cells of melanocytes [18, 19], and are expressed at a higher level in Met-low cells. *Oca2* and *Hps1* promotes melanogenesis *via* melanosome transport [20, 21], and these are expressed at a higher level in Met-high cells. *Xpa* and *Brca2* play a role in nucleotide excision repair [22, 23], which suggests a DNA repair function in UV-sensitive unpigmented cells in Met-low populations. Collectively, these gene expression profiles indicate that Met-low are more melanoblastic, while Met-high are more differentiated to a certain extent.

We confirmed the expressions of several genes by quantitative RT-PCR (Figure 2C). The *Kit* genes play a critical role in the migration and survival of melanoblasts. *Sox10, Pax3* and *Mitf* genes are key regulators of melanocyte development [19, 24], and regulate Met expression in melanocytes and melanoma cells [15, 25]. The expressions of *Kit* and *Sox9* genes were higher in Met-low than in Met-high cells, whereas no significant difference was seen in the expressions of *Mitf* (Figure 2C), *Sox10* and *Pax3* genes (not shown). Consistently, the dual analysis of the cell surface Met and Kit indicated the cells were composed of two major populations characterized by Kit-high/Met-low and Kit-low/Met-high (Figure 2D).

Next, we addressed the sensitivity of Met-low and Met-high cells to the cytotoxic anticancer drugs cisplatin (DNA cross-linking drug) and dacarbazine (DNA alkylating drug), because xenobiotic transporters *Abcb1* and *Abcg2* genes known to cause multi-drug resistance [26, 27] were higher in the Met-low population than in the Met-high population (Figure 2E). Unfractionated cells were cultured for either 3 or 7 days in the absence or presence of 10 μM cisplatin or 1 mM dacarbazine, and surviving cells were analyzed for cell-surface Met expression (Supplementary Figure S1A). In the presence of cisplatin, the Met-high population was clearly decreased and had largely disappeared after 7 days. The same result was obtained for cells cultured in the presence of dacarbazine. The populations of dead cells and apoptotic cells increased following treatment with either cisplatin or dacarbazine, and the numbers were higher in Met-high cells than in Met-low cells (Supplementary Figure S1B). Thus, compared with Met-high cells, Met-low cells were more resistant to chemotherapeutic agents, and the higher expressions of *Abcb1* and *Abcg2* in Met-low cells could have participated in this resistance.

### Higher angiogenic and growth properties in Met-low populations

**Figure 3:**
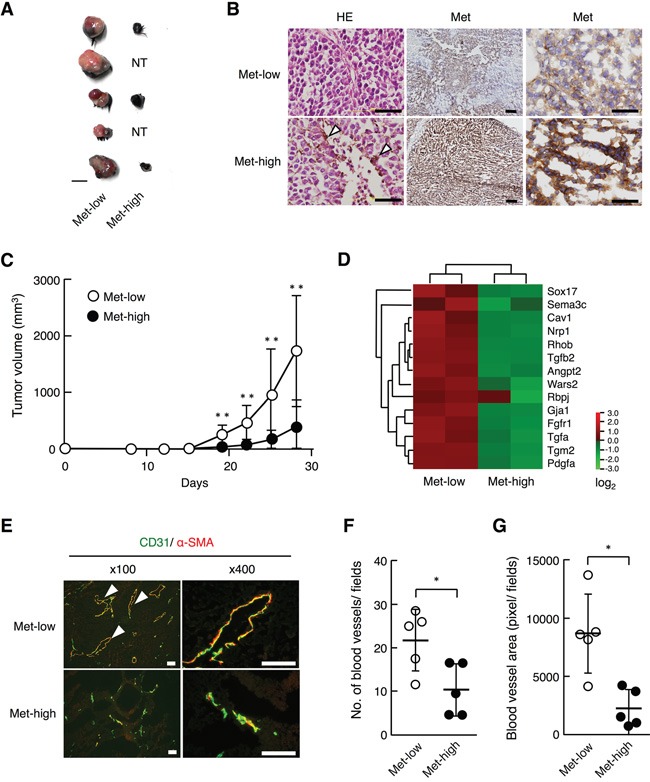
Tumorigenicity and tumor growth of Met-low and Met-high cells subcutaneously implanted in syngenic mice **A.** Appearances of tumors. Tumors developed from Met-low (left side) (n = 11/11) and Met-high (right side) (n = 8/11) cells in the same mouse are indicated in pairs. NT, no tumor. A scale bar, 10 mm. **B.** Histological appearance and Met expression in subcutaneous tumors. Arrowheads indicate pigmented cells. Scale bars, 50 μm in left and right panels; 200 μm in middle panels. **C.** Growth of tumors derived from Met-low (n = 11) and Met-high (n = 11) cells. In independently performed same experiment, substantially the same results were obtained. Each value represents the mean ± SD. **p < 0.01 by Student's *t*-test. **D.** Expression profile of genes characteristic to “blood vessel morphogenesis” in Met-low and Met-high cells. **E.** Blood vessels in subcutaneous tumors, as evaluated by immunohistochemistry for CD31 (green) and α-smooth muscle actin (α-SMA) (red). Arrowheads indicate vascular structures. Scale bars, 50 μm. **F**, **G.** Blood vessel density (F) and area of blood vessels (G). The blood vessel density and area were determined using immunohisotochemical data of individual tumors from Met-low (n = 5) or Met-high (n = 5) cells. The area for luminal structures closed by CD31-positive endothelial was measured by image analysis. *p < 0.05 by Student's *t*-test. In independently performed same experiment, substantially the same results were obtained.

To examine the tumor characteristics of Met-low and Met-high cells *in vivo*, Met-low and Met-high cells obtained after cell sorting were inoculated in the left and right subcutaneous regions, respectively, per each of the C57BL/6 syngenic mice. Both Met-low and Met-high cells formed tumors at a rate of 100%, when subcutaneously inoculated at 100 cells/site (n = 4). However, Met-low cells formed tumors more efficiently than Met-high cells when subcutaneously inoculated at 10 cells/site (100% (11/11) in Met-low cells and 72.7% (8/11) in Met-high cells). Tumors from Met-high cells were highly pigmented, while the tumors from Met-low cells were mostly non-pigmented with the exception of partially pigmented regions (Figure 3A). Immunohistochemical analysis of Met expression showed that Met expression levels were maintained in tumor tissues after 28 days post-inoculation (Figure 3B). Tumor cells derived from Met-low populations showed a low level of Met expression, while tumor cells derived from Met-high populations showed a high level of Met expression.

The tumor volume in the Met-low cells became much larger than in the Met-high cells 28 days post-inoculation (Figure 3C), indicating a more rapid growth potential in Met-low than in Met-high cells. We noticed a higher expression of genes characterized as “blood vessel morphogenesis” and “regulation of angiogenesis” in Met-low cells than in Met-high cells (Figure 3D, Supplementary Table S3), and, therefore, we analyzed tumor-associated angiogenesis by immunohistochemical staining of CD31-positive endothelial cells and α-smooth muscle actin-positive pericytes. The vascular structures of the endothelial cells surrounded by pericytes are abundant in Met-low tumors, whereas the vascular structures of endothelial cells were poorly surrounded by pericytes in Met-high tumors (Figure 3E). The blood vessel density was higher in Met-low than in Met-high tumors (Figure 3F), and the vascular areas surrounded by endothelial cells were much larger in Met-low than in Met-high tumors (Figure 3G) (*P* < 0.05, Mann-Whitney's test). These results suggest that the formation of functional blood vessels may be attributable to the higher growth potential of tumors from Met-low cells.

To know whether selective suppression of Met might influence tumor growth properties, Met-high cells were subjected to the stable expression of shRNA targeting Met (sh-Met) or non-targeting shRNA (Non-target). The knockdown of cell-surface Met expression was confirmed by flow cytometry (Supplementary Figure S2A). When Met-low and Met-high cells (Met-high, sh-Met or Non-target) were inoculated in the left and right subcutaneous regions, respectively, Non-target, sh-Met, and Met-high cells all showed lower tumor growth than that in Met-low cells (Supplementary Figure S2B). These results indicate that a change in Met expression alone does not regulate the difference in the tumor growth properties between Met-low and Met-high cells.

### Highly metastatic properties of Met-high populations to the lung

**Figure 4:**
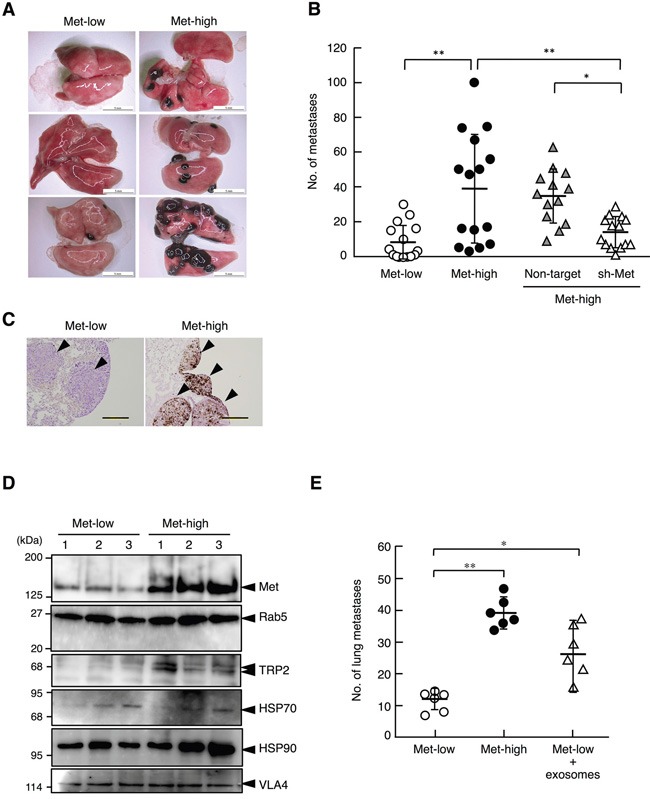
Lung metastasis of Met-low and Met-high cells injected into tail vein **A.** Appearance of metastases in lungs. Scale bars, 5 mm. **B.** The number of metastases in lungs. Met-low (n = 15), Met-high (n = 15), and Met-high cells expressing non-target shRNA (Non-target) (n = 13) or Met-targeting shRNA (sh-Met) (n = 14) were used. **p < 0.01 and *p < 0.05 by Tukey's test. In independently performed three sets of same experiments using a smaller number of animals (n = 2 - 6 for each experimental group), substantially the same results were obtained, and values were combined. **C.** Different pigmentation in metastatic tumors in the lung. Scale bars, 200 μm. **D**, Protein levels in exosomes derived from Met-low and Met-high cells. Protein levels were analyzed by Western blot and Rab5 was used as markers to indicate the amount of exosomes. Substantially same results were obtained in an independently performed experiment using independently prepared exosomes. **E.** The effect of Met-high cell-derived exosomes on metastasis of Met-low cells to the lung. Mice were injected with saline or Met-high cell-derived exosomes for three weeks. Met-low cells were injected two weeks after the treatment with exosomes and lung metastasis was analyzed three weeks post-inoculation. n = 6 in each experimental group. **p < 0.01 and *p < 0.05 by Tukey's test.

We next addressed whether Met-low and Met-high populations might show different metastatic potentials. Met-low and Met-high cells isolated after cell sorting were cultured for 3 weeks (or 6 weeks in the case of cells expressing shRNA, see below), then 10^5^ cells per mouse were injected into the tail veins (n = 13–15 for each experimental group), and the incidences of metastasis to the lungs were analyzed 21 days later. The number of lung metastasis was much higher in Met-high cells (39.1 ± 31.4 per mouse) than in Met-low cells (8.3 ± 10.2 per mouse) (Figures 4A, 4B) (*P* < 0.01, Tukey's test). Histological analysis indicated that tumors from Met-low cells were not pigmented, whereas tumors from Met-high cells were highly pigmented (Figure 4C). The number of lung metastases was significantly suppressed in Met-high cells expressing sh-Met but not in Met-high cells expressing Non-target (Figure 4B). Thus, Met contributes to the lung metastasis of melanoma cells.

A previous study indicated that Met in exosomes released from B16-F10 melanoma cells induces a premetastatic niche formation in lungs, thereby facilitating lung metastasis of melanoma cells [16]. Therefore, we prepared exosomes and analyzed Met protein levels in exosomes derived from Met-low and Met-high cells. Exosomes obtained from Met-low and Met-high cells showed an average size of 110.3 and 112.9 nm in diameter, respectively (Supplementary Figure S3A). The zeta potential values for exosomes from Met-low and Met-high cells were −35.1 and −31.1 mV, respectively, suggesting slightly different electrostatic characteristics (Supplementary Figure S3B). In Western blot analysis, similar Rab5 protein levels indicated that similar amounts of exosomes were released from Met-low and Met-high cells (Figure 4D). In addition to the Met level, the levels of TRP2, HSP70, HSP90, and VLA4 were analyzed, because exosomes from subjects with advanced-stage malignant melanoma contained higher levels of these proteins [16]. The exosomes prepared from Met-high cells contained much higher Met protein than those prepared from Met-low cells. TRP2 and HSP90 were found in larger amounts in the exosomes in Met-high cells than in Met-low cells. Furthermore, the number of lung metastases of Met-low cells was increased to 2.2-fold higher levels by the pretreatment of mice with exosomes derived from Met-high cells (Figure 4D). These data strongly suggest that the Met in exosomes contributes to the lung metastatic potential of Met-high cells.

**Figure 5:**
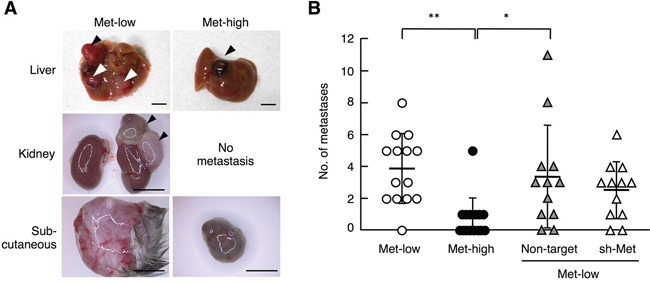
Metastasis of Met-low and Met-high cells to organs other than lungs **A.** Appearance of metastases in the liver, kidney, and subcutaneous (Sc) tissue. Arrowheads indicate tumors. Scale bars, 5 mm. **B.** The number of metastases in various tissues except lungs. Met-low (n = 14), Met-high (n = 15), and Met-high cells expressing non-target shRNA (Non-target) (n = 12) or Met-targeting shRNA (sh-Met) (n = 12) were used.**p < 0.01 and *p < 0.05 by Tukey's test. In independently performed three sets of same experiments using a smaller number of animals (n = 2 - 6 for each experimental group), substantially the same results were obtained, and values were combined.

In addition to lung metastasis, we found that Met-low and Met-high cells showed different metastatic potentials to organs other than the lungs (Figures 5A, 5B, Supplementary Table S5), though the overall absolute number of metastases was much smaller compared with lung metastasis. Met-low cells formed several metastases in the liver, kidney, and subcutaneous tissues (3.9 ± 2.2 per mouse), whereas Met-high cells showed either no metastasis or only a small number of metastases in the liver and subcutaneous tissues (0.7 ± 1.3 per mouse). The metastases derived from Met-low cells were not pigmented, while metastases from Met-high cells were (Figure 5A). The number of metastases of Met-low cells was not significantly changed by shRNA expression targeting Met (Figure 5B, Supplementary Table S5). Thus, Met-low populations include a small number of subfractions, which have metastatic potential for organs other than the lungs. Taken together, these results indicate a clear difference between Met-low and Met-high cells with respect to organ tropism in metastasis and Met is functionally involved in highly metastatic potential to the lungs in Met-high cells.

### Hierarchical phenotypical changes from Met-low into Met-high populations

**Figure 6:**
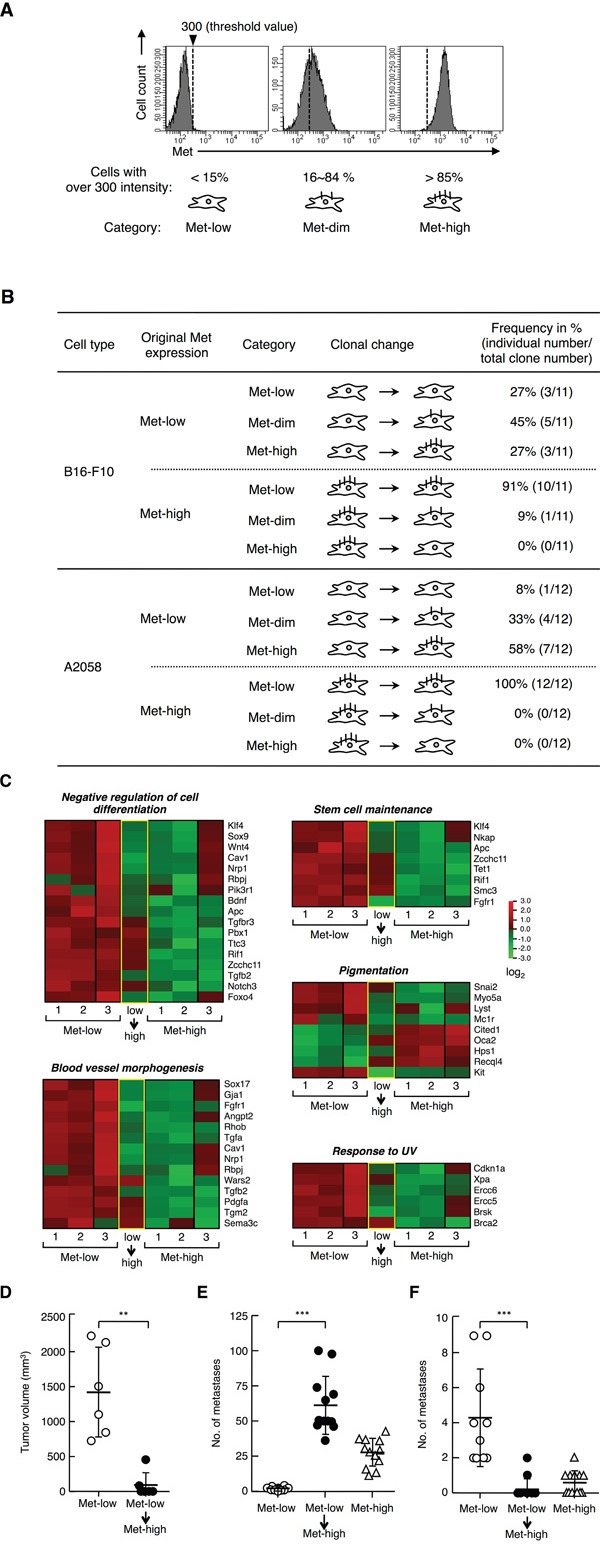
Clonal change in Met expression, gene expression profile, and tumor characteristics of Met-low and Met-high populations **A.** Representative histographical profiles defined as “Met-low,” “Met-dim,” or “Met-high.” The threshold value for fluorescence intensity for Met expression was set at 300, and the cells were divided into 3 groups: “Met-low,” more than 85% of the total cells show a fluorescent intensity of less than 300; “Met-high,” more than 85% of the total cells show a fluorescent intensity of more than 300; and, “Met-dim,” intermediate cells between Met-low and Met-high. **B.** Change in Met expression during clonal expansion of B16-F10 and A2058 cells. Met-low and Met-high cells were sorted by flow cytometry, individually subjected to the clonal growth from a single cell, and cell-surface Met expression was analyzed in each population 21 days (B16-F10) or 28 days (A2058) later. Eleven (B16-F10) or twelve (A2058) clonal cell populations derived from Met-low and Met-high cells were analyzed. Met-low or Met-high clonal cells were obtained by independently performed cell sorting in twice. **C.** Comparison of gene expression profiles between Met-low, Met-high, and the Met-high cells transitted from Met-low (low→high). Among three samples in Met-low or Met-high, the data for two samples (lane 1 and 2) were taken from the same data sets used in Figure 2A and 3D. **D.** Tumor growth. Tumor growth by Met-low cells (n = 6) and the transitted Met-high cells (Met-low→Met-high) (n = 6). Met-low and transitted Met-high cells were inoculated in the same mouse in pairs. *p < 0.01 by Mann-Whitney's test. **E**, **F.** Metastasis to the lungs (E) and organs other than the lungs (F). Met-low (n = 10), Met-high (n = 12), and the transitted Met-high (Met-low→Met-high) (n = 12) cells were used. ***p < 0.0001 and **p < 0.01 by Tukey's test. The experiment was independently performed for three times (n = 2 - 4 for each experimental groups), substantially similar results were obtained, and values were combined.

Next, we addressed whether each population is in a static state or in dynamic equilibrium. Met-low and Met-high cells were sorted by flow cytometry, subjected to clonal growth from a single cell, and the cell-surface Met expressions were analyzed in each population 21 days later (Figure 6, Supplementary Figure S4). The Met expression was divided into 3 groups according to the following categories (Figure 6A): “Met-low,” more than 85% of the total cells show a fluorescent intensity of less than 300; “Met-high,” more than 85% of the total cells show a fluorescent intensity of more than 300; and, “Met-dim,” intermediate cells between Met-low and Met-high.

Of the 11 clonal populations derived from Met-low single cells, 3 populations stayed in Met-low, 5 populations were changed to transitional, and 3 populations were changed to Met-high (Figure 6B, Supplementary Figure S4). In contrast, of the 11 clonal populations derived from Met-high cells, one population was changed to Met-dim, and 10 populations stayed in Met-high. Thus, the Met-low populations were composed of both self-renewing clones and clones changeable from Met-low to Met-high cells. In contrast, most of the Met-high populations were stable, and no clonal population was changed from Met-high to Met-low. These results indicate the hierarchical phenotypic changes from Met-low into Met-high populations.

We next addressed whether the hierarchical change in Met expression could be observed in human malignant melanoma cells. Met expression in A2058 melanoma cells was relatively heterogeneous, and a re-analysis of Met-low and Met-high cells after cell sorting indicated that Met-low and Met-high cells existed as different populations (Supplementary Figure S5A). These populations were subjected to clonal growth, and the cell-surface Met expression was analyzed 28 days later (Figure 6A, Supplementary Figure S5B). Among the 12 clonal populations derived from Met-low cells, one clonal population stayed in Met-low, whereas 11 clonal populations changed to Met-dim or Met-high. In contrast, all 12 clonal populations derived from Met-high stayed in Met-high. Thus, the hierarchical phenotypical changes from Met-low into Met-high populations were observed not only in B16-F10 mouse melanoma cells but also in A2058 human malignant melanoma cells.

A previous study indicated that HGF facilitates resistance to BRAF inhibitor in malignant melanoma cells with an oncogenic BRAF mutation [3, 4]. HGF enhanced the survival of A2058 cells harboring a mutant BRAF in the presence of BRAF inhibitor (vemurafenib) in both Met-low and Met-high cells (Supplementary Figure S6A), and higher survival/resistance was seen in Met-high cells than in Met-low cells at varying concentrations of vemurafenib (Supplementary Figure S6B). The increases in dead and apoptotic cells by vemurafenib were larger in Met-low cells than those in Met-high cells (Supplementary Figure S6C). Thus, the higher Met activation status in Met-high cells than in Met-low cells facilitates resistance to BRAF inhibitor.

Finally, we examined whether clonal transition from Met-low to Met-high cells might be accompanied by a change in tumor characteristics. The Met-high cells that had clonally transitted and expanded from Met-low cells (low→high) were subjected to analyses for a gene expression profile, tumor growth and metastasis. Microarray analysis indicated that the expression of the characteristic genes in the transitted cells showed a transitional profile or closer profile to that of Met-high cells than to Met-low cells (Figure 6C). In subcutaneous tumor growth assays, tumor growth in the transitted cells was much less than that in the Met-low cells (Figure 6D), which is consistent with tumor growth characteristics in Met-low and Met-high cells (Figure 3C). Following intravenous injection, the transitted cells and Met-high cells formed much larger numbers of lung metastases, compared with the Met-low cells (Figure 6E). Met-low cells formed several metastases in organs other than the lungs, whereas the transitted and Met-high cells formed no, or only a few, metastases in organs other than the lungs (Figure 6F, Supplementary Table S6). Thus, the clonal transition from Met-low to Met-high was accompanied with the changes in gene expression profiles, tumor growth, and metastasis.

## DISCUSSION

Melanomas are highly heterogeneous tumors, but the mechanisms for a phenotypic change remained mostly unknown [28]. Analysis of patient-derived xenograft models have shown that phenotypical heterogeneity is reversible within advanced malignant melanoma cells [5, 6]. Consistently, we found that phenotypical heterogeneity characterized by Met expression level is dynamically changed in a cell-intrinsic manner. However, this phenotype change is hierarchical from Met-low to Met high populations. Considering the stem-like gene expression profiles in Met-low populations and the pigmented phenotype in Met-high populations, the cell-intrinsic and hierarchical change from Met-low cells to Met-high cells could be driven by a program reflecting melanocyte development, and this might explain the phenotypic diversity of some cases of melanomas.

Previous studies have indicated that Met functions in cancer stem cells in several cancer cell types, such as colon cancer, prostate cancer, and glioblastoma [29–31]. HGF enhances the epithelial-mesenchymal transition [9–11]. SOX9 participates in the cancer stem cell state and maintains mesenchymal features in some cancers [32]. In the present study, we found that Met is rather expressed in melanoma cells characterized by more differentiated phenotypes such as pigmentation, lower Kit and Sox9 expression. This discrepancy may relate to the particular developmental characteristic of melanocytes, i.e., Met is not expressed in melanoblasts, while the Met expression is induced during melanocyte development by *Mitf* genes [15, 25].

The metastatic characteristics of Met-low and Met-high melanomas differed in organotropism. Met-high cells metastasize mostly to the lungs, whereas Met-low cells metastasize to the liver, kidney, and other tissues. The low frequency of multi-organ metastasis of Met-low cells suggests that only a small population of Met-low cells can metastasize to multiple organs. Identification and characterization of theses cells could lend a better understanding of the multi-organ metastatic potential of melanomas. On the other hand, our results indicate that the highly metastatic potential of Met-high cells functionally required Met expression. Consistent with the previous report describing that exosome-packaged Met from B16-F10 melanoma forms a pre-metastatic niche in lungs [16], we found that exosomes derived from Met-high cells contained much higher levels of Met than those from Met-low cells (Figure 4D), and the number of lung metastases of Met-low cells was increased by the pretreatment of mice with exosomes derived from Met-high cells (Figure 4E). These data strongly suggested that the Met in exosomes functionally contributed to the lung metastatic potential of Met-high cells.

Tumor-associated angiogenesis differs between Met-low and Met-high melanomas. Tumors from Met-high cells developed vascular structures without pericytes. This might be explained by the low expression of platelet-derived growth factor (PDGF) and angiopoietin-2 (Angpt2), because the former facilitates the recruitment of pericytes and the latter induces sprouting angiogenesis [34, 35]. On the other hand, Met-low cells expressed proangiogenic factors at a higher level and tumors from Met-low cells developed more vascular networks surrounded by pericytes. This vascularization in Met-low tumors may function more normally and can support a larger growth potential of Met-low tumors. Instead, the vascular structures lacking pericytes in Met-high tumors may allow transendothelial movement of tumor cells, thereby facilitating intravasation/extravasation of tumor cells during metastatic colonization.

Malignant melanoma is one of the most chemoresistant tumors, and why melanomas are particularly insensitive to chemotherapeutic agents remains poorly understood [36, 37]. Xenobiotic transporters ABCB1 (also known as MDR1 or P-glycoprotein) and ABCG2 (also known as breast cancer resistance protein) plays a major role in drug resistance against chemotherapeutic agents [26, 27, 38, 39]. The higher expression levels of ABCB1 and ABCG2 in Met-low cells may explain, at least in part, why Met-low cell populations are more resistant to chemotherapeutic agents than Met-high cell populations. On the other hand, malignant melanoma harboring a mutant BRAF responds to RAF inhibitors, but the tumors often recur within a certain period of treatment [3, 39]. Expression of HGF and activation of Met confers resistance to BRAF inhibitors in patients [3, 4]. Met-high cell populations show a higher survival rate compared with Met-low cell populations in the presence of BRAF inhibitor (Supplementary Figure S6). Thus, Met-low and Met-high populations have shown resistance to chemotherapeutic and molecular-targeted agents in different ways, in which progenitor cell characteristics and Met activation participates in drug-resistance.

Finally, cell-intrinsic and hierarchical change in Met expression and the associated gene expression profiles could be a mechanism that might help explain the intrinsic diversity and plasticity in tumor growth, metastasis, and drug resistance in malignant melanomas. Elucidation of the mechanism by which Met expression in individual cells is regulated overall in a certain equilibrium between Met-low and Met-high cell populations may facilitate further understanding of the intrinsic nature of malignant melanomas.

## MATERIALS AND METHODS

### Cell culture, exosome preparation, shRNA expression, and reagents

Cells were obtained from the American Type Culture Collection. B16-F10 cells were cultured in RPMI1640 medium supplemented with 10% (v/v) fetal bovine serum. A2058 cells were cultured in DME medium supplemented with 10% (v/v) fetal bovine serum and 2 mM glutamine. For analysis of Met expression in clonal cell populations, cells sorted by flow cytometry were first cultured on a 96-well plate at a density of one cell/well, and were then cultured for 3–4 weeks. Mouse Met shRNA sense sequences were obtained from the Broad Institute TRC shRNA library (Table 1). For exosome preparation, the culture supernatant was centrifuged at 500 × g for 10 min, and filtered through a 0.22-μm filter membrane. The filtrate was centrifuged at 20,000 × g for 20 min, and exosomes were collected by centrifugation at 100,000 × g for 70 min. The particle size and the zeta potential of exosomes were analyzed using a ZETASIZER NANO (Malvern Instruments, Worcestershire, UK). Lentiviral vector encoding shRNA were prepared using HEK-293 cells, and cells transfected with the lentiviral vector were cultured in the presence of 0.5–1.0 μg/ml puromycin for more than 7 days. Cisplatin and dacarbazine were obtained from WAKO Pure Chemicals. Vemurafenib and a BCA Protein Assay Kit were obtained from Selleckchem and Pierce Biotechnology, respectively.

**Table 1:** Sense and antisense sequences for shRNA

Purpose	Sense	Anti sense
Non-target	GCGCGATAGCGCTAATAATTT	AAATTATTAGCGCTATCGCGC
sh-Met	CGGGATTCTTTCCAAACACTT	AAGTGTTTGGAAAGAATCCCG

### Flow cytometry and antibodies

Cells were stained using either a rat anti-mouse Met-PE antibody, a rat anti-human Met antibody (eBiosciences, code: 12-8854-82, clone eBioclone 7, or code: 14-8858-80, clone eBioclone 97), or an anti-mouse Kit antibody (eBiosciences, code: 14-1172-81, clone ACK2). Secondary antibodies conjugated with phycoerythrin (PE) or allophycocyanin (APC) were used (Santa Cruz). Isotype controls were used to set the background. Live-cell selection was accomplished using 7-AAD (BD biosciences). Cells were analyzed and/or sorted on a FACS canto II, a FACS Aria II (Becton Dickinson) or with a JSAN cell sorter (Bay Bioscience, Kobe, JAPAN). Cell cycles were determined using a Cell-Cycle Phase Determination Kit (Cayman Chemical, Ann Arbor, MI). Apoptotic cells were stained using an Annexin V-FITC Apoptosis Kit (BioVision, Milpitas, CA).

### Microarray analysis

Microarray analyses were performed using the Whole Mouse Genome (4 × 44k, G4846A) Oligo Microarray, according to the Agilent 60-mer Oligo Microarray Processing Protocol (Agilent Technologies). Total RNA samples (200 ng) were used to prepare Cy3-labeled cRNA using a Low RNA Input Fluorescent Linear Amplification Kit (Agilent Technologies). Fluorescence-labeled cRNAs were purified using an RNeasy RNA Purification Kit (Qiagen Inc., Hilden, Germany). Two independent RNA samples were used to confirm the reproducibility of the microarray analyses. The images were analyzed using Feature Extraction Software (Ver. 10.7.3.1) and GeneSpring GX 11.5 software (Agilent Technologies). Normalization was performed as follows: (i) intensity-dependent Lowess normalization; (ii) data transformation, with measurements set to ≤0.01; (iii) per-chip 75th-percentile normalization of each array; and, (iv) per-gene: normalized to the median of each gene. Genes differently expressed more than twice between Met-low and Met-high populations were selected and used for the gene ontology enrichment analysis. Gene ontology (GO) enrichment analyses were performed using DAVID Bioinformatics Resources 6.7. The raw and processed data were deposited in the Gene Expression Omnibus (GEO) database (access ID: GSE69741).

### Tumor growth and metastasis assay

Five week-old female C57BL/6 mice (SLC, Shizuoka, Japan) were used. Met-low and Met-high cells were prepared via 2 successive cell sortings. For subcutaneous growth, 10, 50, or 100 cells in 50 μl culture medium were mixed with 50 μl of growth factor-reduced matrigel, and subcutaneously injected bilaterally. Tumor volume was analyzed on day 35 or within 35 days after the inoculation unless otherwise specified. For metastasis assay, 10^5^ cells in 100 μl of PBS were injected into the tail veins, and the degrees of metastases were analyzed 21 days later. All animal experiments were performed in accordance with the animal experiments guidelines of Kanazawa University.

### Histological analysis

For immunostaining of Met, frozen sections were fixed with methanol, tissue sections were incubated with PBS containing 3% bovine serum albumin for 30 min, washed with PBS, and incubated overnight with rabbit anti-Met antibody (Santa Cruz, code: sc-162, clone SP-260) in PBS containing 0.5% bovine serum albumin and 0.1% Tween-20 at 4 °C. After washing, sections were incubated with EnVision+ System-HRP Labeled Polymer (DAKO, code: K4002, Glostrup, Denmark). Immunocomplexes were visualized using an IMMPACT DAB kit (VECTOR, code: SK-4105, PE2 6XS, UK) and nuclei were stained with hematoxylin. For blood vessel staining, frozen sections were fixed with methanol, then treated with rat anti-CD31 (BD, code: 557355, clone MEC13.3) and mouse anti-α-smooth muscle actin (DAKO, code: M0851, clone 1A4, Glostrup, Denmark) antibodies were diluted via a Vector^®^ M.O.M.™ Immunodetection Kit (Vector Laboratories, Burlingame, CA). Sections were analyzed using a Biozero BZ-9000 (KEYENCE, Osaka, Japan).

### Quantitative PCR

Total RNA was prepared using Sepasol RNA (Nacalai Tesque, Kyoto, Japan), and RNA were reverse transcribed using Superscript III Reverse Transcriptase and Oligo(dT) primer (Invitrogen). Quantitative PCR reactions were performed in triplicate using SYBR-green dye (Roche Diagnostics) with the 7500 Sequence Detection System (Applied Biosystems, Streetsville, ON, Canada). The transcription level of β-actin was used for normalization. The primer sequences are listed in Table 2.

**Table 2:** Primer sequences for RT-PCR

Genes	Forward	Reverse
*abcg2*	TCGCAGAAGGAGATGTGTTG	TTGGATCTTTCCTTGCTGCT
*actb*	AGCCATGTACGTAGCCATCC	CTCTCAGCTGTGGTGGTGAA
*kit*	ATCCCGACTTTGTCAGATGG	AAGGCCAACCAGGAAAAGTT
*met*	CTACACCCCAGCCCAAACTA	TGAATTTGAGCGATGCTGAC
*mitf*	AGCCAGCAGGTCAGATCCTA	GGTGGGAAAGACATCCAAGA
*sox9*	ATAAGTTCCCCGTGTGCATC	TACTGGTCTGCCAGCTTCCT
*stx3*	TCAGTCACCCAGCATTCAAG	GCCCTTCATATGTGGCATTT

### Western blotting

Western blotting was performed as described elsewhere [40]. Briefly, a PVDF membrane was incubated with 5% bovine serum albumin for 2 h, and then incubated overnight at 4 °C with primary antibodies. After washing 3 times, the membrane was incubated for 1 h with species-specific horseradish peroxidase-conjugated antibodies, and developed with ECL detection reagent (ImmunoStar, Wako Pure Chemical). For detection of Met tyrosine phosphorylation, cell lysate was subjected to immunoprecipitation with mouse anti-Met (code:#3127, clone 25H2) antibody (Cell Signaling Technology, Beverly, MA) and phospho-Met was detected using rabbit anti-phospho-Met (Y1234/Y1235) (code:#3077, clone D26) antibody (1:1000 dilution, Cell Signaling Technology). Immune complexes were recovered with Protein G-Sepharose beads (Zymed Laboratories, South San Francisco, CA). For exosome analysis, antibodies against Met (25H2), Rab5 (rabbit, code:#5347, clone C8B1) (1:1000 dilution, Cell Signaling Technology), TRP2 (rabbit, code: BS3320, clone K89, 1:1000 dilution, Bioworld, Louis Park, MN), HSP70 and HSP90 (mouse, code: SPA-810-D and ADI-SPA-830, clone C92F3A-5 and AC88, 1:1000 dilution, Enzo Life Sciences, Farmingdale, NY), and VLA4 (rat, code: ab25247, clone PS/2, 1:1000 dilution, Abcam, Cambridge, MA, USA) were used. Can Get Signal^®^ (TOYOBO, Osaka, Japan) was used for the dilution of antibodies.

### Statistical analysis

Error bars represent the means ± standard deviation. Mouse experiments were performed in duplicate using at least 4 mice per treatment group. Multiple-group comparisons were analyzed using either a Tukey's test or ANOVA. Two group comparisons were analyzed using either a Student's *t*-test or a Mann-Whitney's U test using Prism 6 software. A *P* < 0.05 was considered significant.

## SUPPLEMENTARY FIGURES AND TABLES











## Abbreviations

HGF: hepatocyte growth factor
PE: phycoerythrin
GO: gene ontology
GEO: gene expression omnibus
ANOVA: analysis of variance
ABC: ATP-binding cassette.
