# Supplementary material for: Different growth and metastatic phenotypes associated with a cell-intrinsic change of Met in metastatic melanoma
Source: Oncotarget. 2016 Sep 23;7(43):70779–93. doi: 10.18632/oncotarget.12221 (PMC5342589; doi:10.18632/oncotarget.12221)
Supplement: Supplementary file 1 [file oncotarget-07-70779-s001.pdf]

Different growth and metastatic phenotypes associated with a cell-intrinsic change of Met in metastatic melanoma

SUPPLEMENTARY FIGURES AND TABLES

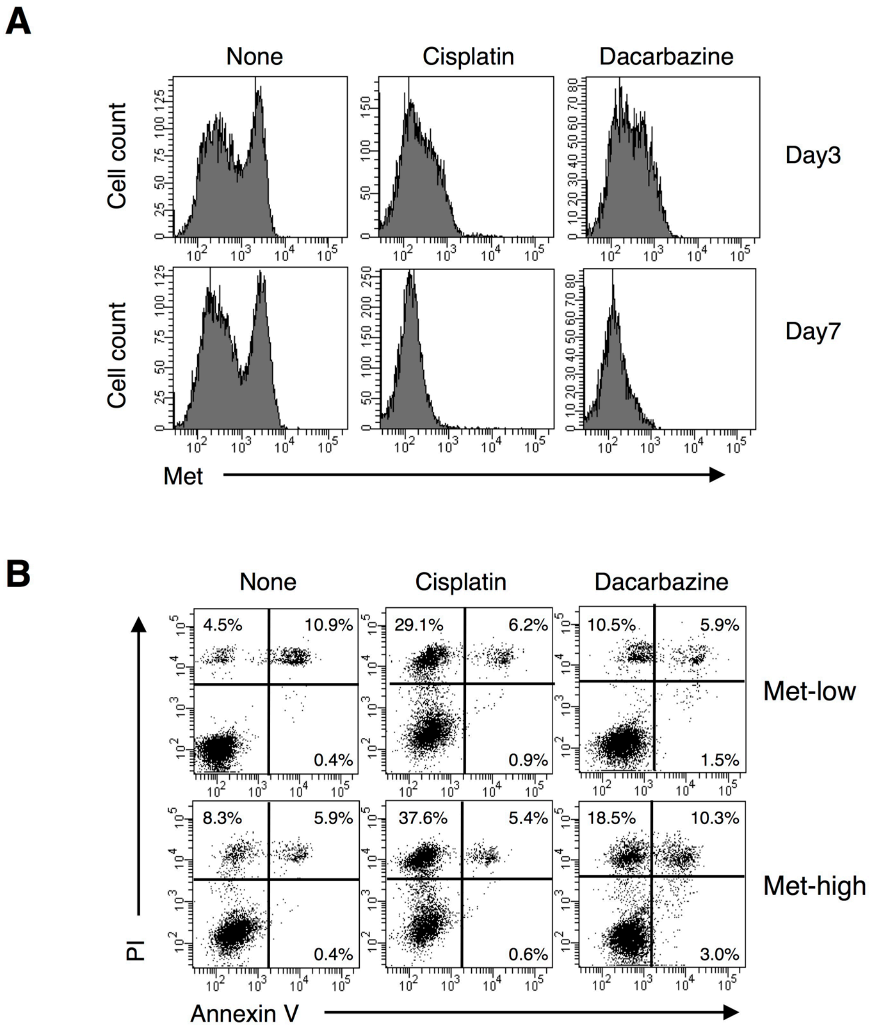

Supplementary Figure S1: Differences in sensitivity of Met-low and Met-high populations to cisplatin and dacarbazine. **A.** Change in Met-low and Met-high populations during culture in the absence or presence of cisplatin or dacarbazine. **B.** Change in distribution of dead cells (PI-positive, Annexin V-negative) and apoptotic cells (Annexin V-positive) in cells with/without 10 µM cisplatin or 1 mM dacarbazine. In A, unfractionated B16-F10 cells were cultured in the absence or presence of 10 µM cisplatin or 1 mM dacarbazine for 3 or 7 days, and viable cells were analyzed for Met expression by flow cytometry. In B, Met-low and Met-high cells were cultured for 48 h, stained with PI and Annexin V, and subjected to flow cytometry. The same experiment was independently performed twice and substantially the same results were obtained.

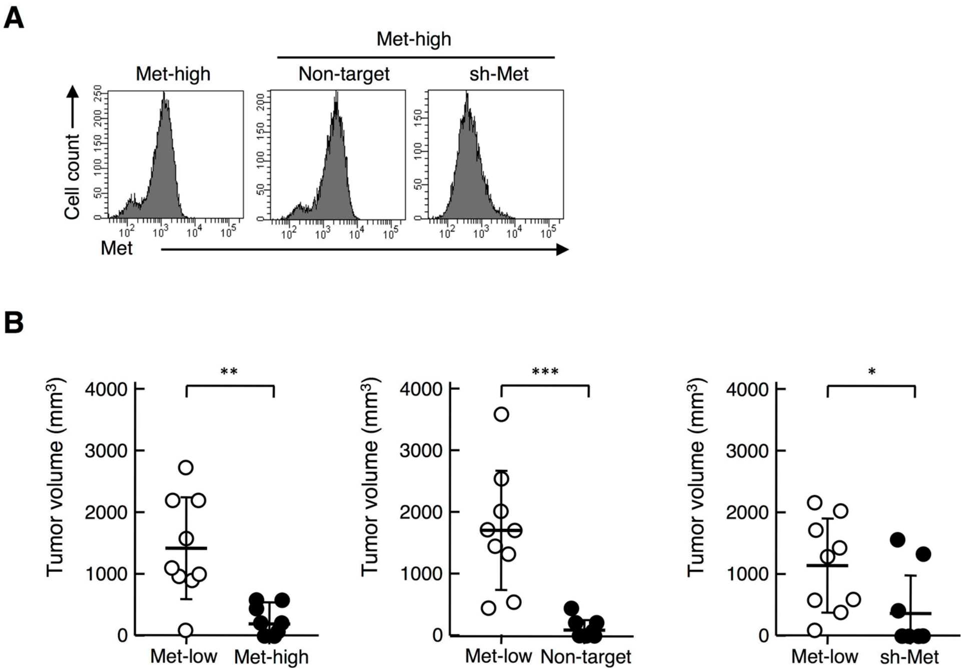

Supplementary Figure S2: Suppression of Met expression by stable expression of shRNA and change in tumor growth potential. A. Cell-surface Met expression in Met-high cells; Met-high cells expressing non-targeting shRNA (Non-target), and Met-high cells expressing Met-targeting shRNA (sh-Met). Met expression was analyzed by flow cytometry. **B.** Growth of tumors derived from Met-low or Met-high (Met-high, Non-target, or shMet) cells. Met-low (n = 9 for each 3 groups, n = 27 in total), Met-high (n = 9), Non-target (n = 9), or sh-Met (n = 9) cells were inoculated at 50 cells/site in the right side in pairs with Met-low cells (left). Each value represents the mean ± SD. ***$p < 0.0001$, **$p < 0.001$, and *$p < 0.05$ by Mann-Whitney's test.

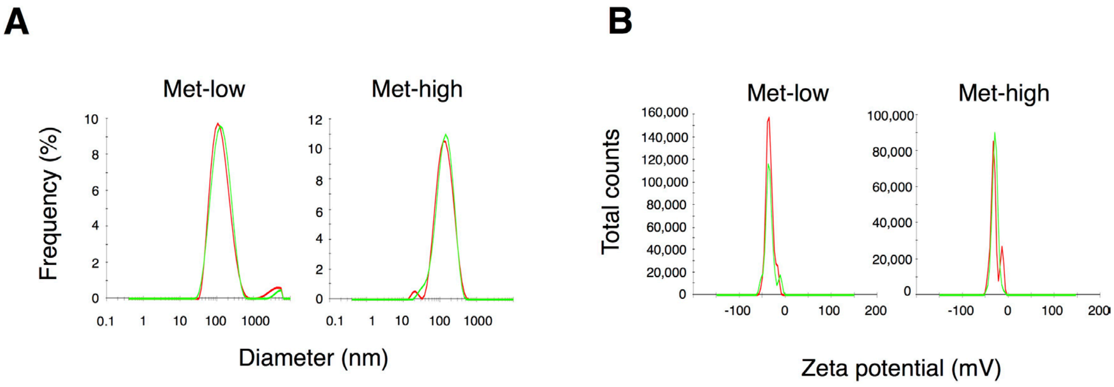

Supplementary Figure S3: Size distribution A. and Zeta potential **B.** of exosomes.

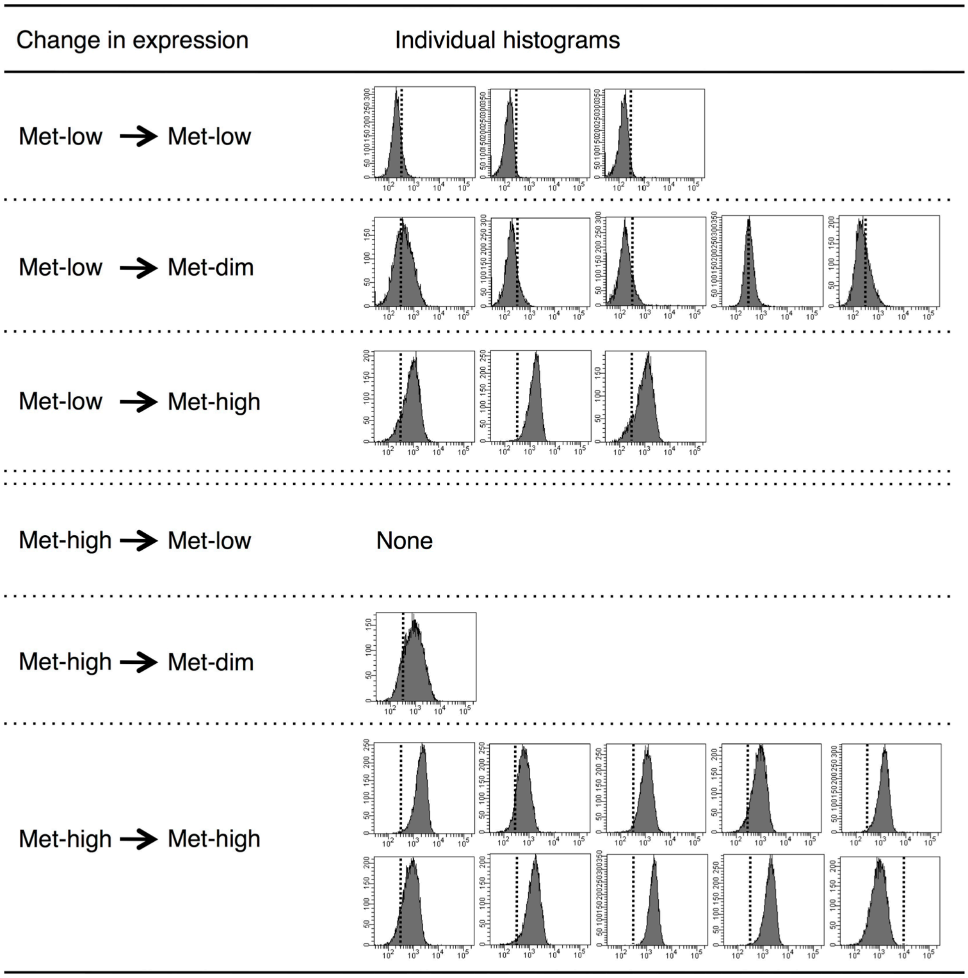

Supplementary Figure S4: Change in Met expression during clonal expansion of B16-F10. Met-low and Met-high cells were sorted by flow cytometry, individually subjected to the clonal growth from a single cell, and cell-surface Met expression was analyzed in each population 21 days later.

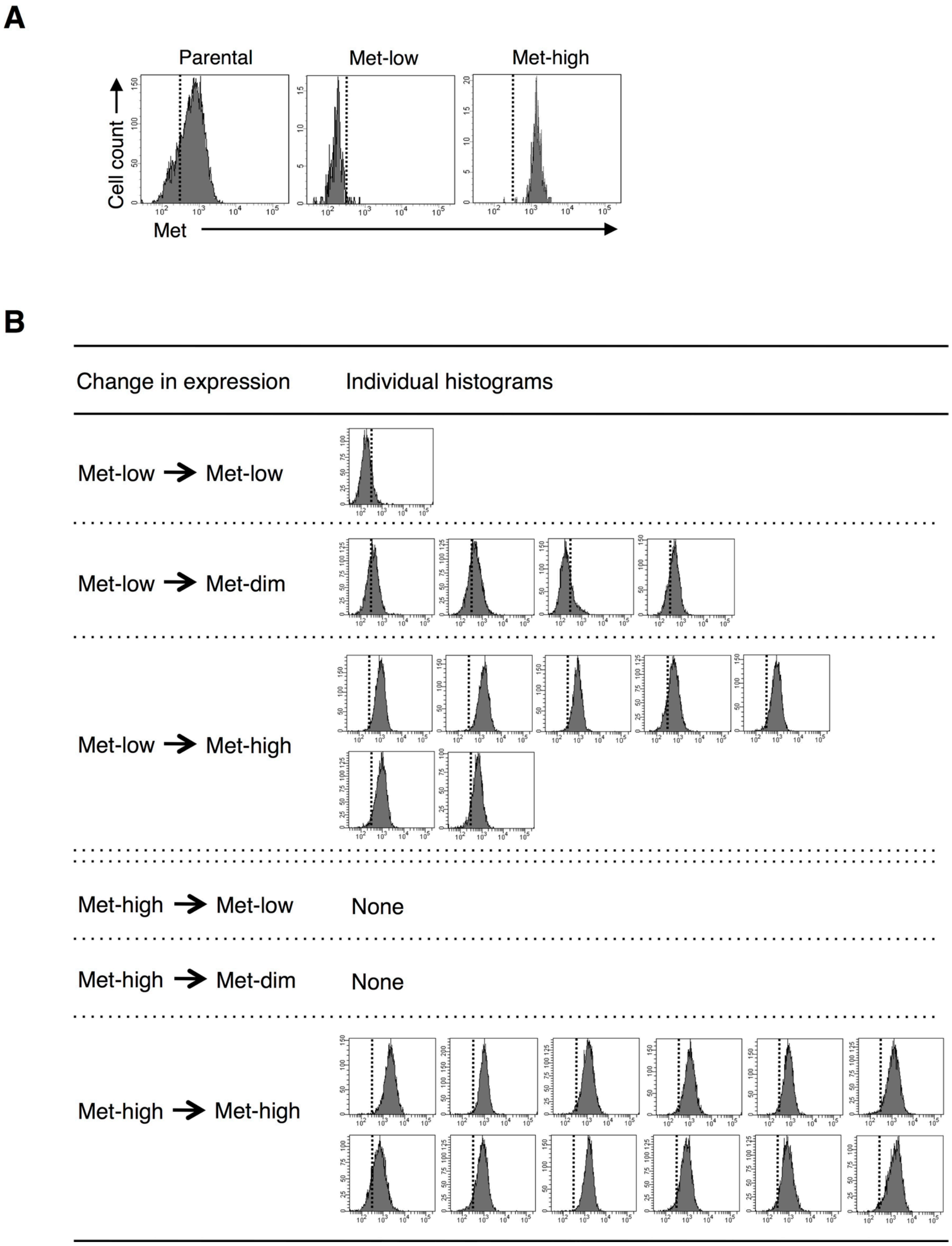

Supplementary Figure S5: Cell-surface Met receptor expression and clonal change in Met expression in A2058 human malignant melanoma cells. A. Cell-surface Met receptor expression and re-analysis of Met expression in Met-low and Met-high cells after cell sorting. **B.** Change in Met expression during clonal expansion. Met-low and Met-high cells were sorted by flow cytometry, individually subjected to the clonal growth from a single cell, and cell-surface Met expression was analyzed in each population 28 days later.

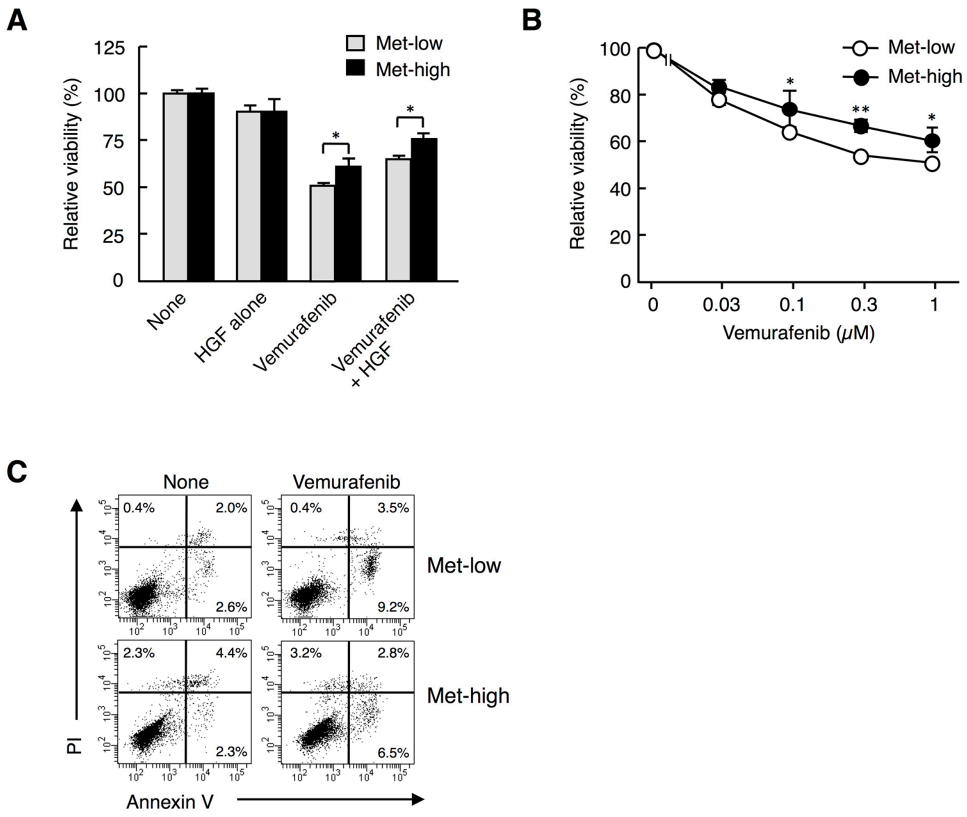

Supplementary Figure S6: Differences in sensitivity of Met-low and Met-high populations to vemurafenib (BRAF inhibitor). A. Sensitivity of Met-low and Met-high populations to vemurafenib in the absence or presence of HGF. Met-low and Met-high cells were cultured in the absence or presence of 1 μM vemurafenib and/or 100 ng/ml HGF for 3 days, and viable cells were evaluated by calcein-AM staining. **B.** Dose-dependent response to vemurafenib. The cells were cultured for 3 days. **C.** Change in distribution of dead cells and apoptotic cells. The cells were cultured for 3 days, stained with PI and Annexin V, and subjected to flow cytometry. In A and B, each value represents the mean ± SD. The assay was performed twice and substantially similar results were obtained. *$p < 0.05$ and **$p < 0.01$ by Student's *t*-test.

Supplementary Table S1: Genes expressed at higher levels in Met-low than Met-high cells

See Supplementary File 1

Supplementary Table S2: Genes expressed at higher levels in Met-high than Met-low cells

See Supplementary File 2

Supplementary Table S3: Gene ontology terms characterizing Met-low cells

See Supplementary File 3

Supplementary Table S4: Gene ontology terms characterizing Met-high cells

See Supplementary File 4

Supplementary Table S5: The number of metastases in organs other than lungs.*

Experimental group	liver	Kidney	adrenal gland	ovary	peritoneal cavity	thymus	brain	lymph node	sub-cutaneous	muscle	bone
Met-low	1.2 ± 1.7	0.3 ± 0.5	0.3 ± 0.5	0.3 ± 0.5	0.9 ± 1.1	0	0.1 ± 0.3	0.1 ± 0.3	0.2 ± 0.4	0.1 ± 0.3	0.7 ± 1.2
Met-high	0.1 ± 0.3	0	0.1 ± 0.4	0.1 ± 0.4	0.3 ± 0.8	0	0	0.1 ± 0.3	0.1 ± 0.3	0	0
non-target	0.7 ± 0.9	0.3 ± 0.5	0.2 ± 0.4	0.2 ± 0.6	0.8 ± 1.4	0.3 ± 0.5	0	0.2 ± 0.4	0.8 ± 1.0	0	0.1 ± 0.3
sh-Met	0.1 ± 0.3	0.5 ± 0.9	0.3 ± 0.6	0.4 ± 0.7	0.6 ± 1.4	0	0	0.1 ± 0.3	0.4 ± 0.8	0	0.2 ± 0.6

*The values indicate the number of metastases per mouse in each organ. Each value represents the mean ± SD.

Supplementary Table S6: The number of metastases in organs other than lungs.*

Experimental group	liver	kidney	adrenal gland	ovary	peritoneal cavity	thymus	brain	lymph node	sub-cutaneous	muscle	bone
Met-low	0.7 ± 1.1	0.3 ± 0.5	0.5 ± 0.7	0.1 ± 0.3	1.2 ± 0.6	0.8 ± 0.8	0	0.2 ± 0.6	0.5 ± 0.7	0	0
Met-low ↓ Met-high	0	0	0.1 ± 0.3	0	0	0.1 ± 0.3	0	0	0.1 ± 0.3	0	0
Met-high	0	0	0	0	0	0.3 ± 0.5	0	0.2 ± 0.6	0.1 ± 0.3	0.1 ± 0.3	0

*The values indicate the number of metastases per mouse in each organ. Each value represents the mean ± SD.
